# Community Transmission *via* Indirect Media-To-Person Route: A Missing Link in the Rapid Spread of COVID-19

**DOI:** 10.3389/fpubh.2021.687937

**Published:** 2021-07-28

**Authors:** Qunfang Hu, Lei He, Ying Zhang

**Affiliations:** ^1^Shanghai Institute of Disaster Prevention and Relief, Tongji University, Shanghai, China; ^2^College of Architecture and Urban Planning, Tongji University, Shanghai, China; ^3^College of Environment and Resource, Fuzhou University, Fuzhou, China

**Keywords:** COVID-19 pandemic, community transmission, indirect transmission route, media-to-person, contaminated residential environment

## Abstract

To prevent the spread of coronavirus disease 2019 (COVID-19), stringent quarantine measures have been implemented so that healthy people and virus carriers have isolated themselves in the same community owing to the limit capacity of healthcare facilities. With the exponential growth of the infected population, the residential environment is contaminated by fomites from the infected residents and consequently threating the health of susceptible residents. Till now, little has been acknowledged on this indirect transmission route and its role on community transmission. Here we address the impact of self-isolated virus carriers on the residential environment and elucidate the potential transmission pathways *via* contaminated environment in communities. We urge further investigation on the superspreading cases in communities and hope to arouse the attention to evaluate the potential risk of indirect transmission route as well as the corresponding control measures.

## Introduction

The outbreak of coronavirus disease 2019 (COVID-19) induced by sever acute respiratory syndrome coronavirus (SARS-CoV-2) is the most significant global public health emergency in the twenty-first century, which has been characterized as a pandemic by WHO on March 11, 2020. SARS-CoV-2 is highly infectious with the estimated basic reproduction number *R*_0_ = 2 – 3.5 and thus rapidly expanding to more than 200 countries and territories in <4 months since the first reported case in China in December, 2019 (1, 2). Facing the threat of a new infectious disease, quarantine is the oldest but the most effective approach to control its spread. Almost all the countries across the world have taken the stringent measures such as “stay-at-home,” “social-distancing,” and “lock-down” to control and prevent the rapid spread of COVID-19. However, due to the limit capacity of medical facilities and the self-limiting nature of COVID-19, healthy people and infected individuals such as patients with mild to moderate symptoms obey to isolate themselves at home in the same community. So far, little has been acknowledged on the role of the mixed quarantine of healthy and infected people together in the spread of COVID-19, but the risk from contaminated environment exists (3). Considering the exponential increase of the infected population and the high risk of the potential transmission in communities, we address the possible transmission routes of COVID-19 in the high-density residential communities based on the current understanding of SARS-CoV-2.

## The Lifetime of SARS-CoV-2 in the Environment and Its Transmission Routes

Together with SARS-CoV-2, there are now seven coronaviruses causing respiratory and gastrointestinal infections in human being. SARS-CoV, MERS-CoV, and SARS-CoV-2 are highly pathogenic to humans whereas HCoV-OC43, HCoV-229E, HCoV-HKU1, and HCoV-NL63 merely induce mild infection in immunocompetent persons (4). SARS-CoV-2 probably originating from bats and infecting humans *via* an unknown intermediate host, is transmittable between animals and humans like other coronavirus (5). What worthy noting is that SARS-CoV-2 is highly contagious even during the latency period. Similar to other coronaviruses, SARS-CoV-2 can invade the respiratory and gastrointestinal systems. Based on the clinical reports, the gastrointestinal symptoms could precede the typical respiratory ones. Besides, some researches point out the possible transmission *via* body fluid or maternal-neonatal route, but these results are not conclusive now (1).

Since human coronavirus infection is regarded as a mild, self-limiting disease, little has been known about its viability, infectivity, and transmission potential outside the host. Generally, coronavirus could survive several hours in aerosol or on the dry surface, while they persist for days in water, especially at water temperature <4°C (vital for >300 days) (6, 7). Table 1 summarized the stability of SARS-CoV and SARS-CoV-2 in aerosol, solid surface and water. Both SARS-CoV and SARS-CoV-2 survived for hours in aerosol or on the solid surface (8). Although the stability of SARS-CoV-2 in water is still unknown, SARS-CoV with strong genome similarity to SARS-CoV-2 was reported to remain viral for days in water and wastewater (6). More recently, SARS-CoV-2 has also been detected in sewage from public facilities (9), such as airports, the designated hospitals, and hotels for quarantine. The exact concentration of viruses in water or wastewater needs further investigation, but virus loads are high in feces (SARS-CoV in stool sample around 10^7^ gc/mL) and even comparable to that in saliva in some cases (10, 11). Thus, SARS-CoV-2 seems to survive for a long period after excreted from patients to the environment and consequently increasing the possibility of direct or indirect pathways to transmit COVID-19 *via* human excretion, fomites, or contaminated environmental media.

**Table 1:** Survival time of coronavirus in environmental media.

**Virus**	**Aerosol (8)**	**Solid surface** **(** **8** **)**				**Water** **(** **5** **)**		
		**Plastic**	**Stainless steel**	**Copper**	**Cardboard**	**Type**	**4^**°**^C**	**20–25^**°**^C**
SARS-Cov	3 h	72 h	72 h	8 h	8 h	Tap water	>14 d	2–3 d
						Normal saline	>14 d	>14 d
						Feces	>17 d	3–17 d
SARS-Cov-2	3 h	72 h	72 h	4 h	24 h	NA	NA	NA

**Figure 1:**
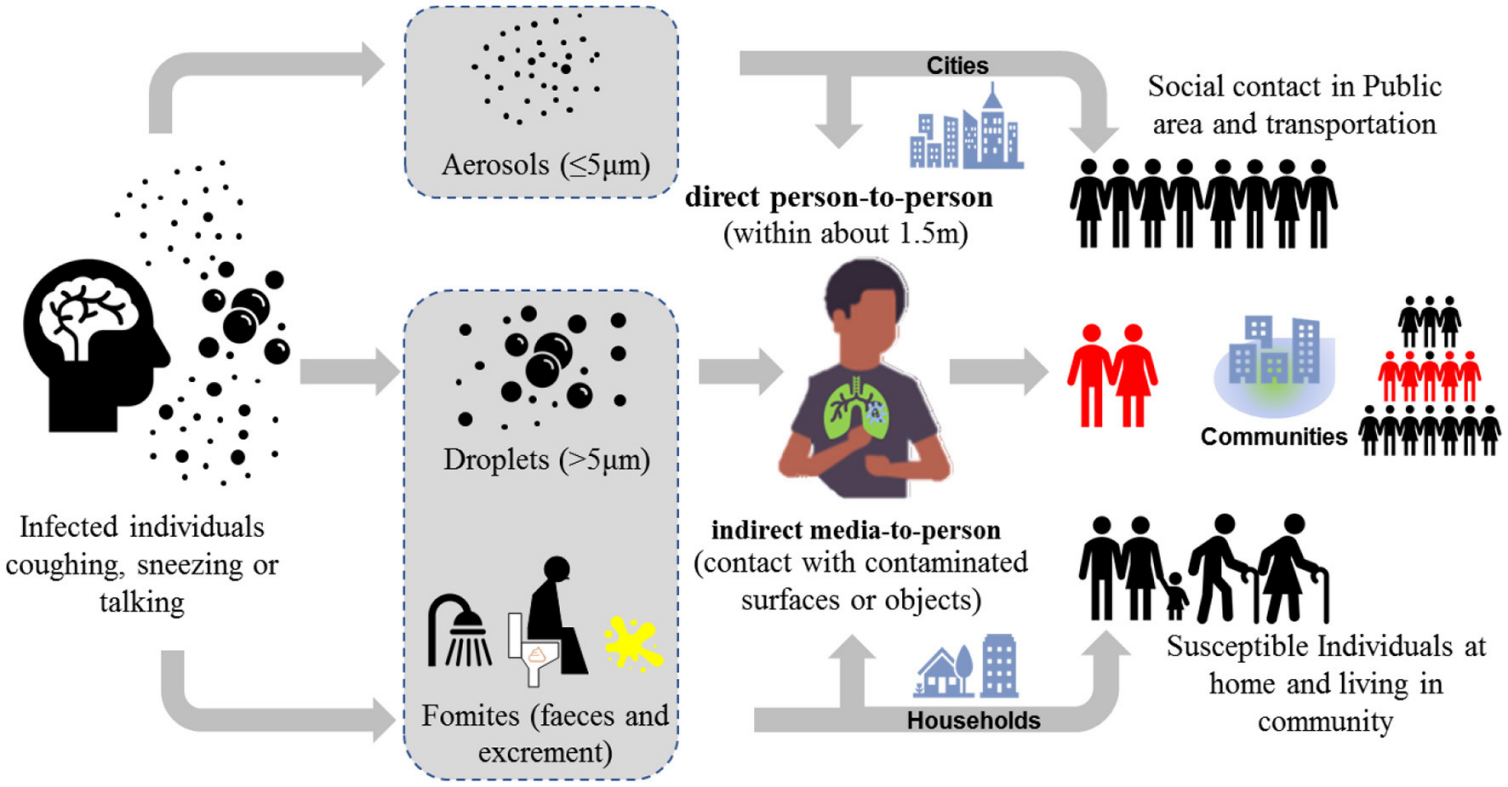
Transmission routes *via* two modes (1) direct person-to-person and (2) indirect media-to-person of COVID-19.

Based on the special characteristics of SARS-CoV-2, there are several direct and indirect transmission routes such as person-to-person, fecal-oral, and airborne route (Figure 1). Person-to-person transmission *via* respiratory droplets and close contact is dominated, but the indirect transmission routes (i.e., media-to-person) also plays a critical role in the spread of COVID-19. The primary pathways of indirect transmission include the inhalation of aerosols with SARS-Cov-2 (i.e., airborne route) and the contact of contaminated environment such as public facilities (i.e., fecal-oral route). The reported infection cases of children reinforced the possibility of fecal-oral transmission route *via* contaminated environment (12), while the sewage and ventilation systems are suspected to contribute in the dramatic spread of COVID-19 on the Diamond Princess cruise ship. In fact, during the outbreak of SARS in 2003, a large clusters of infection at Amoy Garden in Hong Kong has already suggested feces from patients with diarrhea could contaminate the residential environment through sewage and ventilation systems and thus causing cluster transmission *via* airborne route in the housing complex (13). Besides, the COVID-19 resurgence in several provinces in China recently indicates that contaminated cold-chain food is a possible origin of COVID-19 outbreak as well (14). Since SARS-Cov-2 survives in environment (especially in cold and wet condition) for days, it is of critical importance to elucidate and clarify the role of these secondary transmission pathways, though they may be previously regarded as elusive and opportunistic.

Moreover, the discovery of the virus shedding of asymptomatic infected individuals (15) arouses broad attention to the transmission of SARS-CoV-2 from asymptomatic persons to healthy people, since a substantial fraction of COVID-19 cases are asymptomatic or mild symptoms. According to Chinese Center for Disease Control and Prevention (CCDC), 81% of the cases had mild symptoms and 1.2% were asymptomatic among 72,314 confirmed cases in China. COVID-19 exhibits a long incubation period of 1–14 d and could be contagious during the latency period (1, 2). Because the vial loads of asymptomatic and symptomatic individuals were similar, healthy people may probably get infected by asymptomatic or presymptomatic individuals. Although the intensity and range of the asymptomatic transmission has not been addressed in the spread of COVID-19, various infection cases by asymptomatic or presymptomatic individuals have already been reported in China, Japan, and the United States. Besides, domestic animals like cats might play a key role as intermediate hosts that enable the transmission of SARS-Cov-2 from contaminated environmental media to human being, since animals *per se* also suffer disease caused by coronaviruses. The possibility of this routine has increased after the finding that SARS-CoV-2 infected cats as well (16). Nonetheless, there is no direct evidence showing the transmission from domestic animals like cats and dogs to human being and thus requiring further investigation.

## Community Transmission *via* Direct and Indirect Routes

**Figure 2:**
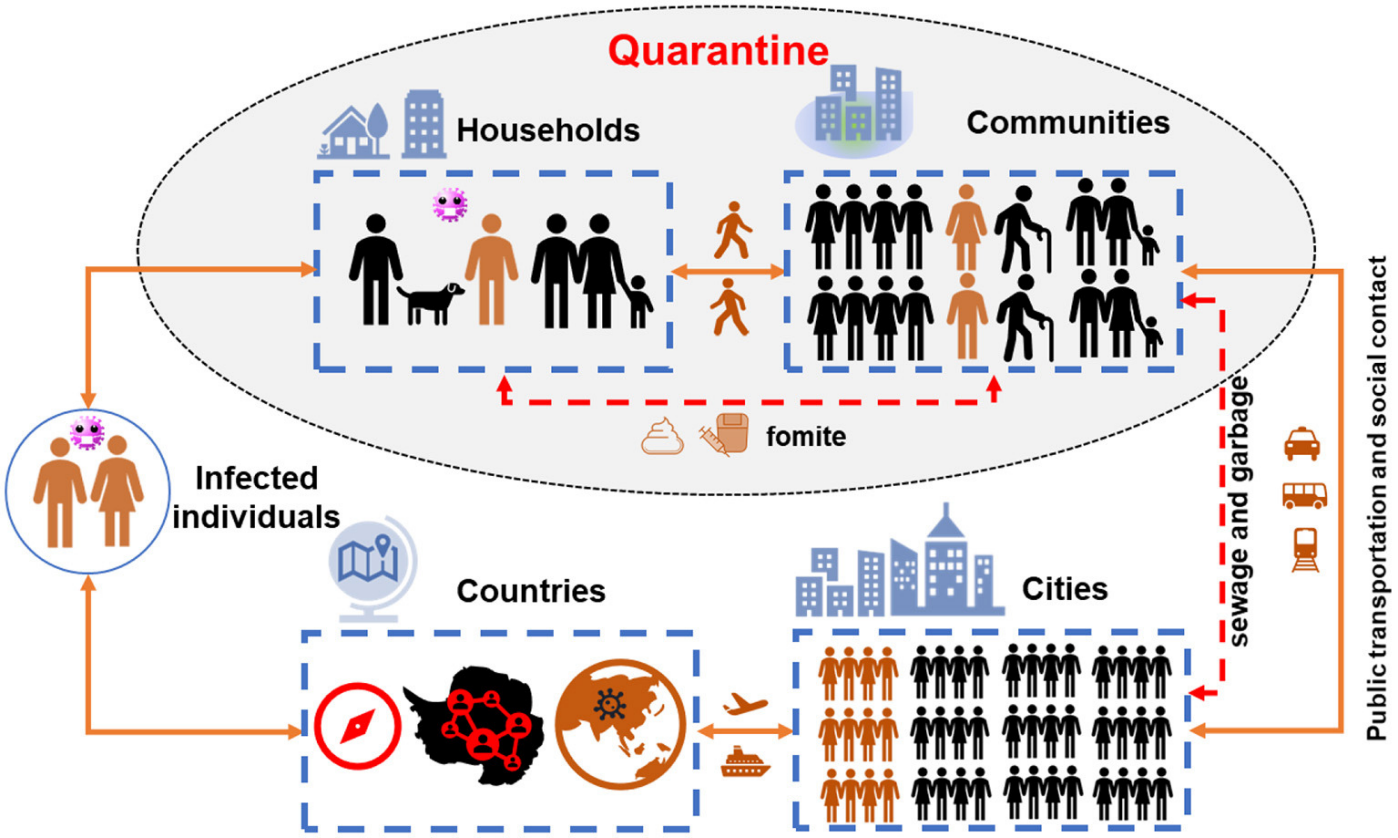
The transmission chain of COVID pandemic. The yellow lines show the trajectory of infected individuals migrating from households to countries *via* public transport (direct person-to-person pathway), while the red dash lines indicate the municipal infrastructure to convey wastewater and garbage in urban area (the indirect media-to-person pathway). The shaded part shows the potential community transmission during quarantine.

**Figure 3:**
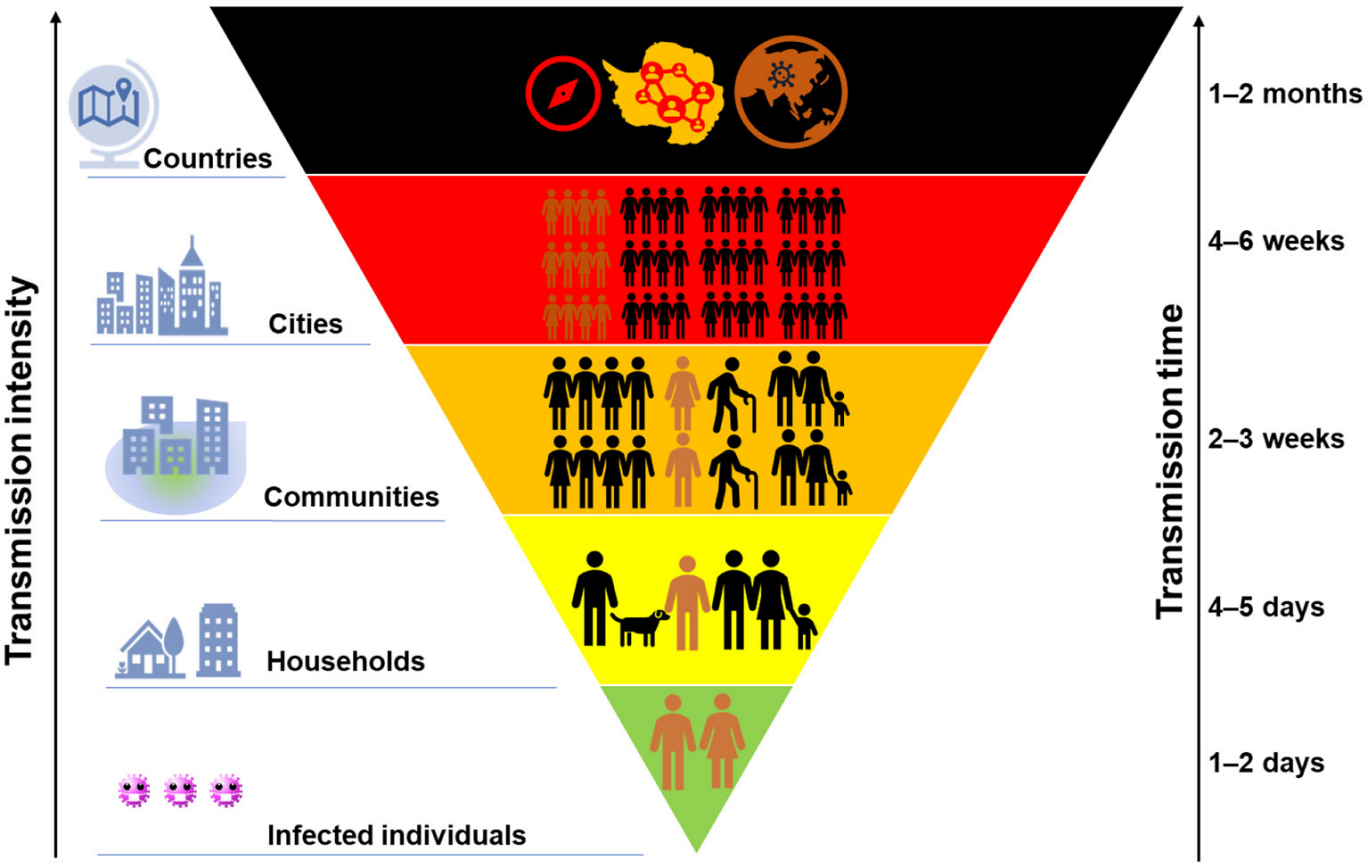
A schematic of the intensity of COVID-19 transmission with time. The size of colored block indicates the transmission scale of COVID-19, while its background color from green to dark suggests the severity.

Figure 2 demonstrates the transmission chain of SARS-CoV-2 without any containment strategies, i.e., infected individual–household–community–city–country. Due to the rapid person-to-person transmission of SARS-CoV-2, COVID-19 became pandemic in 1–2 months (Figure 3). To control and prevent the spread of COVID-19, containment strategies have been implemented to suppress person-to-person transmission, which is the primary route to transmit the disease. Many cities shut down public facilities, suspended public transport, and request residents self-isolating and telecommuting to avoid sporadic cases circulating in the city to spread the disease (Figure 2). However, this containment strategy postulates that there is no or limited community transmission. If infected individuals (e.g., asymptomatic persons or even patients with mild to moderate and unspecific symptoms) are active in the community before their recovery, it would greatly increase the possibility of community transmission. To better clarify this issue, here we term symptomatic, presymptomatic and asymptomatic persons as virus carriers and classify virus carriers (direct) and the environment (indirect) contaminated by them as two reservoirs of the disease. That is, there are two pathways in community transmission, i.e., direct person-to-person and indirect media-to-person pathway.

After quarantine, virus carriers and healthy people are isolated at home and gathered together in the same community. Primarily, domestic transmission occurs in households *via* direct contact with virus carriers, as previously happened in Wuhan. After that, the residential environment in communities becomes easily contaminated by fomites and fecal excretion from infected residents because of high virus loads in stool (10, 11). With an increase of the infected population, fecal-oral or airborne transmission route becomes possible through sewage and ventilation system in the buildings, as explained previously. Recently, Beijing has already reported infection cases due to the contact with contaminated public facilities such as stairs and elevators in the apartment, which supports our assumption of media-to-person pathway. In other words, susceptible residents such as elders and persons with underlying diseases are vulnerable to COVID-19 through either direct contact with virus carriers (person-to-person pathway) or indirect transmission by the contaminated environment media (media-to-person pathway). If both person-to-person and media-to-person transmission occurs, COVID-19 would rapidly spread in communities and significantly increase the infected population. As a result, a large portion of critical patients who need intensive health care would overdraw the capacity of healthcare facilities and thus making it impossible to “flatten the curve.”

Furthermore, in poor sanitation areas, sewage carrying large amount of viruses may directly discharge into natural water bodies or even invade the source of drinking water (e.g., groundwater) around the community. As a result, humans as well as animals exposed to the contaminated water bodies may get infected, although the infectivity of SARS-CoV-2 in wastewater is supposed to be low. Domestic waste from virus carriers would have a similar health risk to residents and animals as sewage does. In addition, the presence of SARS-CoV-2 in wastewater or waste poses a high health risk to the staff and volunteers who selflessly continue their daily work in the community. Therefore, these indirect transmission routes, which are regarded as less significant and often ignored in the past, may leads to a secondary spread of COVID-19 within communities, while safe drinking water, sanitation, and hygiene can protect healthy residents from any infectious disease, including COVID-19.

Although little has been acknowledged on the potential community transmission, it is reasonable that virus carriers should be identified and separated from healthy people in the community. Unfortunately, limited to the capacity of testing, patients with mild to moderate symptoms and never traveling to severely affected regions barely have a diagnostic test for SARS-CoV-2. Hence, the incidence of community transmission by patients with mild symptoms or asymptomatic virus carriers is still lacking now. Under such circumstance, the mixed isolation of virus carriers and healthy people in the same community may become a big issue to block the transmission of COVID-19. In a word, community transmission *via* direct person-to-person and indirect media-to-person pathway may dramatically challenge and jeopardize the effort of quarantine, especially in the megacity with high-density population and communities such as London, New York and Paris.

## Summary

Based on current evidences, we believe that community transmission has become the hotspot of COVID-19 transmission *via* direct person-to-person and indirect media-to-person pathway in the megacities across the world. We therein presented several potential transmission pathways within communities and addressed the impact of self-isolated virus carriers at households on the surrounding environment in high-density residential community. Despite being underestimated previously, indirect media-to-person transmission of COVID-19 *via* fecal-oral and airborne route has significant implications, especially in areas with clustered communities and the poor sanitation. Here we offer three approaches to interrupt the transmission in communities: (a) intensive and extensive virus test for identifying carriers (symptomatic, presymptomatic and asymptomatic persons), (b) hierarchical isolation and centralized treatment for virus carriers, and (c) disinfection of sewage and garbage to guarantee community hygiene. Given to the potential risk of community transmission, we urge further investigation and evaluation on current superspreading cases to fill the gap in community transmission. We also hope to arouse the attention to assess the current quarantine measure and containment strategies in order to better control and prevent the spread of COVID-19 in communities.

## Data Availability Statement

The original contributions presented in the study are included in the article/supplementary material, further inquiries can be directed to the corresponding authors.

## Author Contributions

All authors listed have made a substantial, direct and intellectual contribution to the work, and approved it for publication.

## Conflict of Interest

The authors declare that the research was conducted in the absence of any commercial or financial relationships that could be construed as a potential conflict of interest.

## Publisher's Note

All claims expressed in this article are solely those of the authors and do not necessarily represent those of their affiliated organizations, or those of the publisher, the editors and the reviewers. Any product that may be evaluated in this article, or claim that may be made by its manufacturer, is not guaranteed or endorsed by the publisher.
